# Daily precipitation dataset at 0.1° for the Yarlung Zangbo River basin from 2001 to 2015

**DOI:** 10.1038/s41597-022-01471-7

**Published:** 2022-06-18

**Authors:** Keke Zhao, Dingzhi Peng, Yu Gu, Bo Pang, Zhongfan Zhu

**Affiliations:** College of Water Sciences, Beijing Normal University, Beijing, 100875 China

**Keywords:** Hydrology, Hydrology

## Abstract

In order to obtain higher precision regional precipitation dataset in the Yarlung Zangbo River basin, two different schemes were proposed on the basis of the two most application potential satellite-based precipitation products, IMERG and CMORPH_BLD. The first method aimed to correct the positive error of IMERG based on high correlation (CC > 0.9) between IMERG and gauges. The second algorithm was developed to merge IMERG with CMORPH_BLD by the stepwise linear regression. As the reference, IMERG played a key role in correction of precipitation ratio determination and precipitation event detection. Two daily datasets with 0.1° resolution (BRD_IMERG and IGREA_IMERG-CMORPH) performed better than IMERG in CC, RMSE, ME, FAR and CSI, and streamflow simulation in the whole basin (NS: 0.86 and 0.87; RBIAS: −19% and −11%) and sub-basins. The two proposed methods were relatively simple and efficient for reconstructing higher precision regional precipitation, and the datasets provided a good application demonstration in the alpine region.

## Abstract (summary)

**Table:** 

Measurement(s)	precipitation
Technology Type(s)	satellite and weather station
Factor Type(s)	CMORPH_BLD • IMERG
Sample Characteristic - Organism	basin
Sample Characteristic - Environment	large alpine basin
Sample Characteristic - Location	Yarlung Zangbo River basin

## Background & Summary

**Fig. 1:**
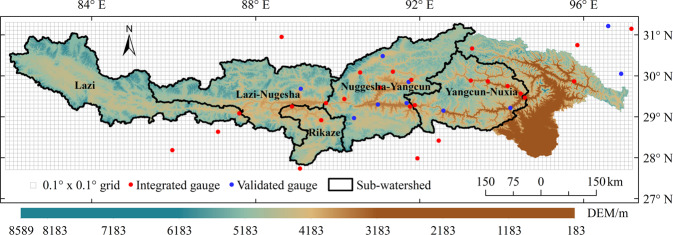
Map of the Yarlung Zangbo River basin.

High precision and resolution precipitation record is essential for hydrological research in a large basin with varying topography and huge differences of elevation. In the Yarlung Zangbo River basin (Fig. 1) of the Tibetan Plateau, precipitation varies strongly in space, however, the gauged stations are sparse and mainly located in relatively low elevation. Satellite precipitation exhibits consistent spatial pattern and seasonal cycle with gauged observations^1^, and could provide more comprehensive of precipitation for modelling studies, but there are still uncertain errors for satellite estimation which is caused by sensor measurements and could be corrected by reanalysis or gauged data^2–6^. The common global satellite precipitation products include: the Precipitation Estimation from Remotely Sensed Information using Artificial Neural Networks (PERSIANN)^7^, Climate Prediction Center Morphing Technique (CMORPH)^3^, Global Satellite Mapping of Precipitation (GSMaP)^5^, the TRMM Multi-Satellite Precipitation Analysis (TMPA)^8^ and Integrated Multi satellite Retrievals for GPM (IMERG)^9^. Due to different retrieval algorithms in productions, their difference and bias are large in different study area^10–13^. The whole basin was divided as five sub-basins (Fig. 1).

Before GPM era, TMPA was normally considered as the most reliable satellite-based precipitation among numerous products and widely used in the research of drought, landslide and flood^11,14–17^. As the successor of TRMM, the constellation-based satellite mission GPM was launched in the early 2014. The key advancement of GPM over TRMM is the extended capability to measure light rain and snowfall^6^. Actually, IMERG were constantly evaluated in different region and compared with other global precipitation products^9,18–24^. It is reasonable to conclude that IMERG outperforms TMPA over Mainland China in most cases and the probability density function (PDF) of IMERG generally match the PDF of gauges. IMERG is better suited for hydrological applications^9,21,23^. Based on combining multiple existing microwave rainfall algorithms and the passive microwaves aboard various spacecrafts^3,25,26^, CMORPH also shows a high applicability in Tibetan Plateau^27,28^. National Meteorological Information Centre of China has utilized the original PDF-optimal interpolation (OI) algorithm to generate the gauge-satellite merged precipitation product by using more than 30,000 gauged data and CMORPH^29,30^. Therefore, the performance of IMERG and CMORPH was worthy of exploration in the Yarlung Zangbo River basin.

Many researchers attempted to reproduce the precipitation dataset in different region with gridded data of global analysis, reanalysis and satellite products. However, the achievement for the Yarlung Zangbo River basin was relative less and mainly focused on the large area of Tibetan Plateau. Maussion *et al*.^31^ and Jiang *et al*.^32^ applied dynamical downscaling method to generate high-resolution precipitation datasets of Tibetan Plateau based on analysis data with coarse resolution and regional climate model. However, the precision of downscaled precipitation data would depend on the key physical processes and accurately parameterized in the models^1^. Also, some researchers aimed to fuse a variety of precipitation products into a high-quality dataset at fine scale based on statistical method^33^, neural network method^2,13,34–36^, interpolation method of OI and PDF-OI^29,30,37,38^ and so on. Specifically, Sun and Su^39^ interpolated gauge-based data to high spatial resolution grids in the Yarlung Zangbo River basin and then corrected the interpolated dataset by the orographic, precipitation gradient and reanalysis dataset GLDAS; Wang *et al*.^40^ and Hong *et al*.^13^ integrated multiple reanalysis and satellite product (ITP-Forcing, MERRA2, TRMM, GSMaP, IMERG, CMORPH and so on) with gauged data in the Yarlung Zangbo River basin and Tibetan Plateau, respectively. The performance of reconstructed datasets really was improved for the chosen reference.

In this study, we tried to propose the relatively simple and efficient methods for reconstructing higher precision regional precipitation dataset in the alpine basin. Section 2 provided the description of evaluation methods, input data and two calibrated frames. Sections 3 described the comparative metric and hydrological evaluation results of two final datasets and one intermediate dataset.

## Methods

### Evaluation

High precision reconstruction precipitation needs reliable source data as input. The six common global daily precipitation products were downloaded and evaluated in the Yarlung Zangbo River basin to obtain reliable source data. These six products are TRMM 3B42 (0.25°)^41^, TRMM 3B42 RT (0.25°)^42^, CMORPH_BLD (0.25°)^29^, GSMaP_Gauge_NRT (0.1°)^43^, PERSIANN-CDR (0.25°)^44^ and IMERG (0.1°)^45^.

The CC^46^ was calculated to show the agreement degree of precipitation product with the observations, and the best value of CC is 1. The CC equation is as below:

1 $$CC=\frac{{\sum }_{\kappa =1}^{\theta }\left({B}_{\kappa }-\bar{B}\right)\left({P}_{\kappa }-\bar{P}\right)}{\sqrt{{\sum }_{\kappa =1}^{\theta }{\left({B}_{\kappa }-\bar{B}\right)}^{2}}\sqrt{{\sum }_{\kappa =1}^{\theta }{\left({P}_{\kappa }-\bar{P}\right)}^{2}}}$$

Where *θ* is the total days; κ stands for the κ*-*th day; *B*_*κ*_ and $\bar{B}$ stand for the observed precipitation and the mean observed precipitation, respectively; and *P*_*κ*_ and $\bar{P}$ stand for the precipitation of product and the mean precipitation of product, respectively.

Error estimation metrics of RMSE and ME^46^ are typical statistical indicators to measure the error and gap between observed precipitation and precipitation product. The best values for RMSE and ME are 0.

2 $$RMSE=\sqrt{\frac{1}{\theta }\mathop{\sum }\limits_{\kappa =1}^{\theta }{\left({P}_{\kappa }-{B}_{\kappa }\right)}^{2}}$$

3 $$ME=\frac{1}{\theta }\mathop{\sum }\limits_{\kappa =1}^{\theta }\left({P}_{\kappa }-{B}_{\kappa }\right)$$

The precipitation events metrics are usually used to measure the detection accuracy of precipitation events of product^18,20,47–49^. The metrics includes the probability of detection (POD, the best value is 1), the false alarm rate (FAR, the best value is 0) and the critical success index (CSI, the best value is 1). POD shows the ratio of precipitation events that were correctly detected while FAR shows the ratio that was actually false alarms. CSI is defined as the function of FAR and POD, which describes the ratio of precipitation events correctly detected by precipitation product among the sum number of precipitation events detected by rain gauge and precipitation product.

4 $$POD=\frac{a}{a+c}$$

5 $$FAR=\frac{b}{a+b}$$

6 $$CSI=\frac{1}{\frac{1}{1-FAR}+\left(\frac{1}{POD}\right)-1}\;or\;CSI=\frac{a}{a+b+c}$$

Where *a* is the number of hit events for which both the precipitation of product and rain gauge detect positive precipitation in total days; *c* is the number of missed events for which the rain gauge detects precipitation but the product records does not in total days; and *b* is the number of false alarms for which the rain gauge detects no precipitation but the record of precipitation show positive precipitation in total days.

The Nash-Sutcliffe efficiency (NS)^50^ and the relative bias (RBIAS)^40^ are classical metrics to assess the performance of driving data in the hydrological model.

7 $$NS=1-\frac{{\sum }_{\kappa =1}^{\theta }{\left({B}_{\kappa }^{r}-{S}_{\kappa }^{r}\right)}^{2}}{{\sum }_{\kappa =1}^{\theta }{\left({B}_{\kappa }^{r}-\overline{{B}^{r}}\right)}^{2}}$$

8 $$RBIAS=\frac{{\sum }_{\kappa =1}^{\theta }{S}_{\kappa }^{r}-{\sum }_{\kappa =1}^{\theta }{B}_{\kappa }^{r}}{{\sum }_{\kappa =1}^{\theta }{B}_{\kappa }^{r}}\times 100 \% $$

Where ${B}_{\kappa }^{r}$ and ${S}_{\kappa }^{r}$ are the observed streamflow and the simulated streamflow by hydrological model in the κ*-*th day, respectively. $\overline{{B}^{r}}$ is the mean observed streamflow.

### Hydrological model

As a large-scale, semi-distributed hydrologic model, the Variable Infiltration Capacity (VIC)^50,51^ contains the snow^52,53^ and frozen soil^54^, which is applicable to the hydrological simulation in the alpine basin. The performance of different precipitation products could be reflected with the simulated streamflow when they were considered as the precipitation driver for VIC.

### Input data

#### GPM IMERG Final Precipitation L3 1-month V06 (GPM_3IMERGM, hereafter refer to as IMERGM)

IMERG algorithms build upon the algorithms included GPCP^55^, PERSIANN^7^, NRL-Blend^56^, SCaMPR^57^, TMPA^4^, CMORPH^3^, and GSMaP^5^, were used to merge microwave precipitation estimation, microwave-calibrated infrared (IR) estimation, precipitation gauge analyses, and potentially other precipitation estimators at fine time and space scales. The monthly IMERGM (0.1° × 0.1°) could be download from the Goddard Earth Sciences Data and Information Services Centre (GES DISC)^58^.

#### GPM IMERG Final Precipitation L3 1-day V06 (GPM_3IMERGDF, hereafter refer to as IMERG)

The half-hour multi-satellite estimation as input data are summed to the monthly scale first and then combined with the monthly GPCC precipitation gauge analysis. Subsequently, the monthly product is used to rescale the half-hourly product and then the daily product is accumulated by the half-hourly estimation. Actually, the monthly rainfall rates of GPM_3IMERGM are equal to the sum value of daily IMERG in each month. The IMERG (0.1° × 0.1°) could be download from GES DISC^45^.

#### CMORPH_V1.0BLD_0.25 deg (hereafter refer to as CMORPH_BLD)

First, CPC Morphing system constructs a purely satellite-based precipitation estimation (raw CMORPH), and then the daily gauged data is used to bias correct the raw CMOPRH through probability density function (PDF), results in a high-resolution global precipitation (bias-corrected CMORPH, 30 min and 8 km × 8 km), and well converted to the CMORPH Climate Data Record (CMORPH_CDR). The Blended Gauge-CMORPH is developed by combining the CMORPH_CDR and the CPC gauge analysis with an optimal interpolation (OI) approach^29^. The daily Blended Gauge-CMORPH (0.25°) was used in the study and could be download from the ftp server of the National Oceanic and Atmospheric Administration (NOAA)/National Center for Environmental Prediction (NCEP)/Climate Prediction Center (CPC)^59^.

### Reference data

#### Rain gauge data

China Meteorological Data Service Centre (http://data.cma.cn) provides multi-time-scale rain gauge data of China, and the Tibet Hydrology and Water Resources Survey Bureau is also responsible for measuring various meteorological data and runoff data. 36 gauges (26 gauges were used to merge with satellite-derived precipitation products and the rest 10 gauges were used to validate the reprocess products in Tables 1 and 2) which located in or around the Yarlung Zangbo River basin (Fig. 1) as the reference records of precipitation.

**Table 1:** Information of rain gauges for calibration (2001~2015).

No.	Gauge	Elevation/m	No.	Gauge	Elevation/m
1	Shenzha	4672	14	Nimu	3809
2	Luolong	3640	15	Nugesha	3700
3	Jiali	4489	16	Yangcun	3600
4	Pangduo	4050	17	Rikaze	3836
5	Yangbajing	4250	18	Zedang	3552
6	Gongbujiangda	3400	19	Lazi	4000
7	Tangjia	3850	20	Jiangzi	4040
8	Baheqiao	3216	21	Dingri	4300
9	Bomi	2736	22	Longzi	3860
10	Gengzhang	3000	23	Nielaer	3810
11	Lasa	3658	24	Cuona	4280
12	Linzhi	3000	25	Pali	4300
13	Nuxia	2910	26	Changdu	3306

**Table 2:** Information of rain gauges for validation.

No.	Gauge	Elevation/m	Time range
1	Gongga	3555	2001–2012
2	Jiacha	3260	2001–2012
3	Dangxiong	4200	2001–2015
4	Nanmulin	4000	2001–2015
5	Milin	2950	2001–2015
6	Langqiazi	4432	2001–2015
7	Basu	3260	2001–2015
8	Qiongjie	3740	2006–2015
9	Mozhugongka	3804	2001–2015
10	Leiwuqi	3810	2001–2015

### Methodology description

**Fig. 2:**
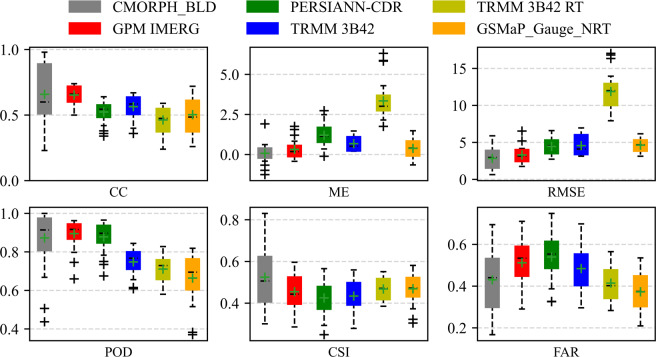
Evaluation results for six satellite-derived precipitation products in the Yarlung Zangbo River basin.

**Fig. 3:**
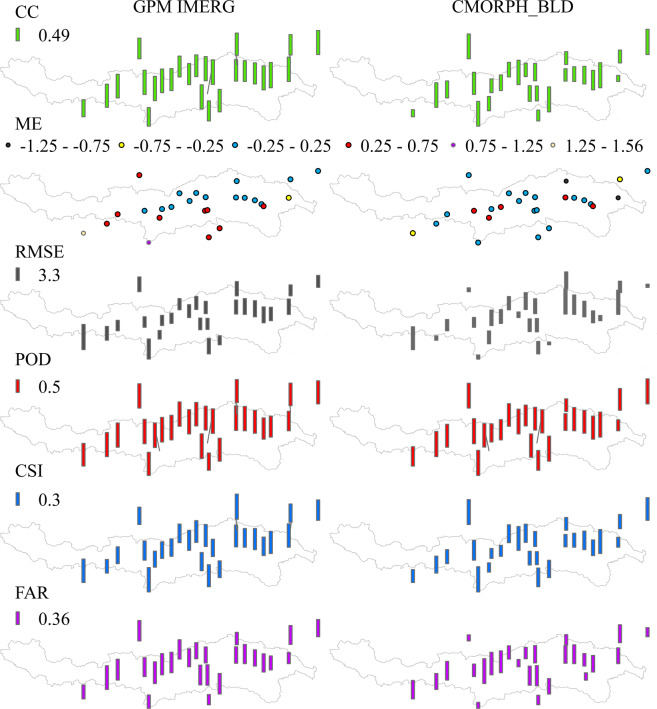
Evaluation results of IMERG and CMORPH_BLD.

Against with 26 gauges, CMORPH_BLD and IMERG showed the highest correlation and the smallest error because of the highest median CCs (0.62 and 0.66), the smallest median MEs (0.07 and 0.18 mm/day) and median RMSEs (2.69 and 3.23 mm/day) in Fig. 2. Overall, the correlation relationship with gauged data for IMERG was good and steady in space, but there was large spatial variability for CMORPH_BLD though it had better correlation for some local site in Fig. 3 (30% of CCs was over 0.8). 83% of MEs for IMERG and CMORPH_BLD were concentrated in the range of (−0.5, 0.5), they could underestimate the precipitation (ME < 0) for nearly the same number of gauges (around 30%). The underestimation extent of CMORPH_BLD was far larger than IMERG (Fig. 2). For PERSIANN-CDR, TRMM 3B42, TRMM 3B42 RT and GSMaP_Gauge_NRT, the median CCs, ME, and RMSE were (0.54, 0.60, 0.47 and 0.50), (1.18, 0.52, 3.01 and 0.45 mm/day) and (4.69, 4.11, 12.00 and 4.68 mm/day), respectively. TRMM 3B42 RT had the worst performance both in correlation and error. In the precipitation event detection, median POD, CSI and FAR for IMERG were 0.92, 0.44 and 0.53, and they were 0.92, 0.51 and 0.44 for CMORPH_BLD. PERSIANN-CDR had high median POD (0.9) but relatively low median CSI (0.42) and FAR (0.56). The median PODs for TRMM 3B42 (0.77), TRMM 3B42 RT (0.73) and GSMaP_Gauge_NRT (0.70) were lower than other three products, and the difference between CSIs and FARs for different products was little. Comprehensively, CMORPH_BLD and IMERG were the two higher precision satellite-based precipitation products compared with others.

**Fig. 4:**
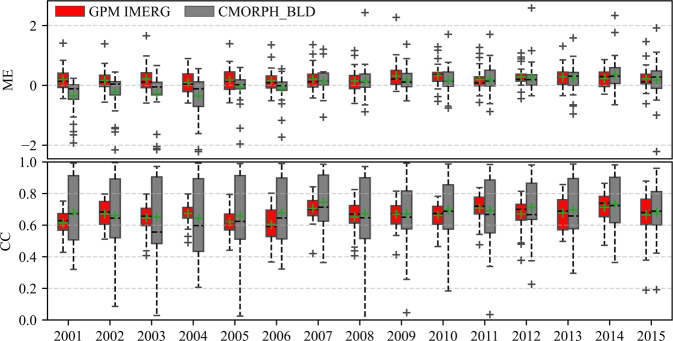
ME and CC of daily precipitation of IMERG and CMORPH_BLD with rain gauge records from 2001 to 2015.

Combined with MEs and CCs from 2001 to 2015 (Fig. 4), CMORPH_BLD showed large spatial variability in most years for ME and all years for CC. CMORPH_BLD underestimated from 2001 to 2006 but overestimated from 2007 to 2015. Small difference in CCs and MEs) revealed that IMERG was more consistent than CMORPH_BLD in time and space.

### Adjusted daily IMERG

**Fig. 5:**
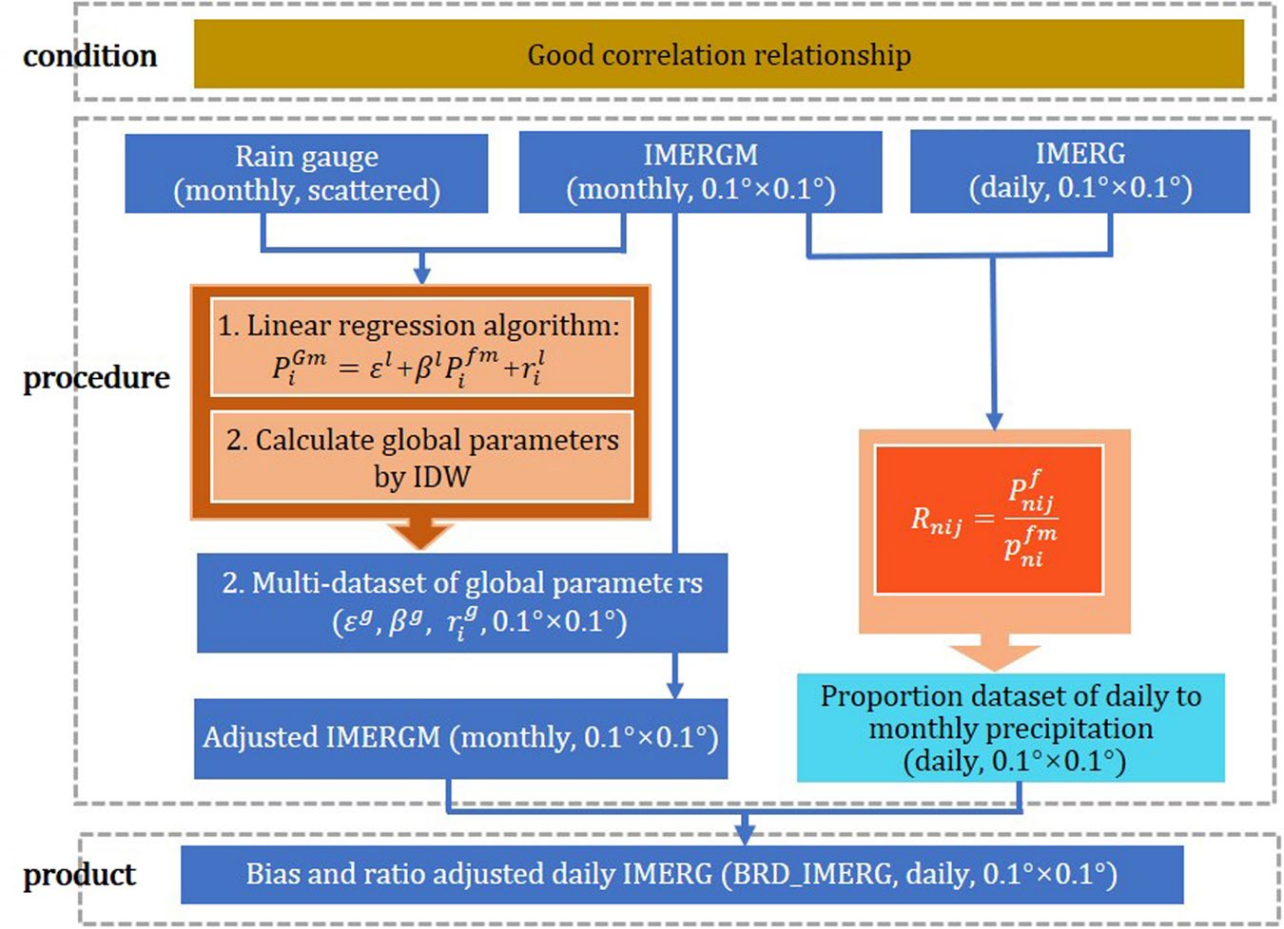
Flow chart of the bias and ratio adjusted daily IMERG dataset. (Note: *n,i* and *j* are year, month and day, respectively; *ε*^*l*^, *β*^*l*^ and ${r}_{i}^{l}$ are the local parameters of linear regression model, which represent the intercept, the coefficient and the monthly residual, respectively; *ε*^*g*^, *β*^*g*^ and ${r}_{i}^{g}$ are the global parameters which were calculated with local parameters by using IDW; *P*^*Gm*^, *P*^*fm*^ and *P*^*f*^ represent the monthly gauged data, monthly IMERGM and daily IMERG, respectively; R is the proportion of daily IMERG to monthly IMERGM.

The first method aimed to correct the positive error and bias of IMERG based on linear correlation relationship between IMERGM and gauged data. The flow chart (Fig. 5) was summarized as follow: first, local parameter combinations (*ε*^*l*^, *β*^*l*^ and ${r}_{i}^{l}$) of linear regression model between gauges and IMERGM were calculated, after getting rid of two extreme parameter combinations, the values of *ε*^*l*^ and *β*^*l*^ were in ranges of (−7, 6) and (0.4, 1.2). Second, the local parameters were interpolated to 0.1° resolution in global basin by using inverse distance weighting (IDW)^60^, and then the global parameters were used to correct IMERGM. At last, the proportion of daily data (IMERG) to monthly data (IMERGM) was used to allocate monthly bias-corrected IMERGM to daily dataset, called the bias and ratio adjusted daily IMERG (BRD_IMERG).

### GPM-CMORPH-Merged dataset

**Fig. 6:**
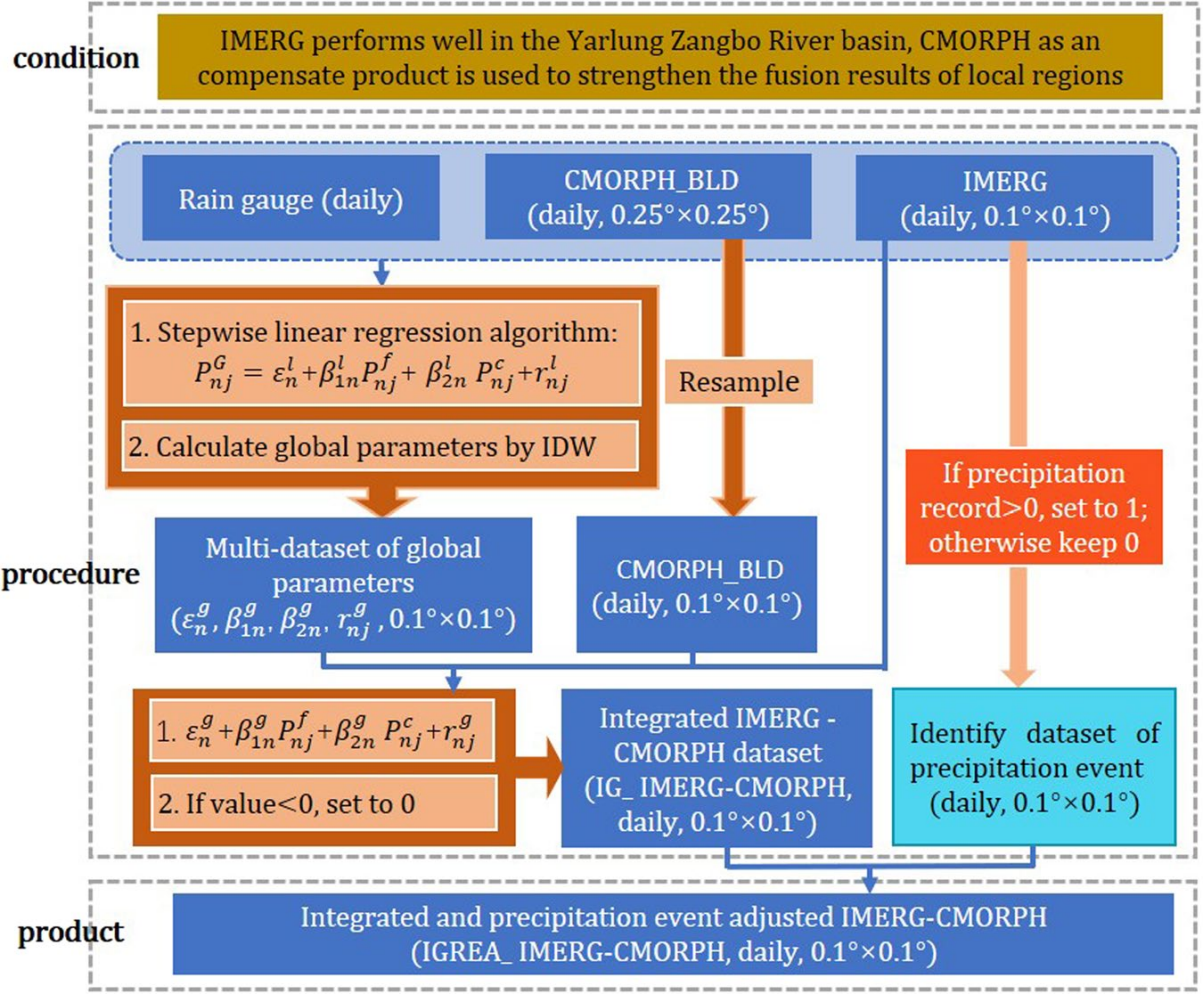
Flow chart of the integrated and precipitation event adjusted IMERG-CMORPH dataset by using CMORPH_BLD, IMERG and gauged data. (Note: ${\varepsilon }_{n}^{l}$, ${\beta }_{1n}^{l}$, ${\beta }_{2n}^{l}$ and ${r}_{nj}^{l}$ are the local parameters of stepwise linear regression model in the *n*-th year, which represent the intercept, the coefficient of IMERG, the coefficient of CMORPH_BLD and the residual, respectively. ${\varepsilon }_{n}^{g}$, ${\beta }_{1n}^{g}$, ${\beta }_{2n}^{g}$ and ${r}_{nj}^{g}$ are the global parameters which were calculated with local parameters by using IDW; *P*^*G*^ and *P*^*c*^ represent the daily gauged data and the daily CMORPH_BLD, respectively.

The second method aimed to merge IMERG and CMORPH_BLD. According to the above analysis, CMORPH_BLD could perform better than IMERG in some gauges for CC, ME and precipitation event detection. Therefore, CMORPH_BLD was considered as the data fusion with IMERG. The stepwise linear regression model was constructed. The values of ${\beta }_{1n}^{l}$ were distributed in (−0.18, 0.93) and 85% was larger than 0. The values of ${\beta }_{2n}^{l}$ were distributed in (−0.4, 1.4), 83% was in (0, 1) and 13% was larger than 1. IDW was used to interpolate local parameters values to global ones. Combined with 0.1° datasets of global parameters, CMORPH_BLD and IMERG, the preliminary integrated dataset (IG_IMERG-CMORPH) could be obtained. Detailed flow chart could be found in Fig. 6. When the daily estimation of reference product IMERG was larger than 0 (or equal 0), the data was re-recorded to 1 (0) in the precipitation event identified dataset which could be used to revise wrong precipitation event of IG_IMERG-CMORPH. The final reconstructed product was called the integrated and precipitation event adjusted IMERG -CMORPH (IGREA_ IMERG-CMORPH).

## Data Records

Two categories of daily precipitation datasets were produced by the flow charts of Figs. 5 and 6, and the raster data with tiff format was uploaded as two zip files. Each zip file consists of two datasets: the final dataset and the intermediate dataset (distinguished by the flow chart). All daily precipitation record (mm) for a 24-hour period starts at 00:00UTC in each day and the data is from 2001 to 2015. The entire archive could be found at figshare^61^.

## Technical Validation

### Evaluation against with gauged data

**Fig. 7:**
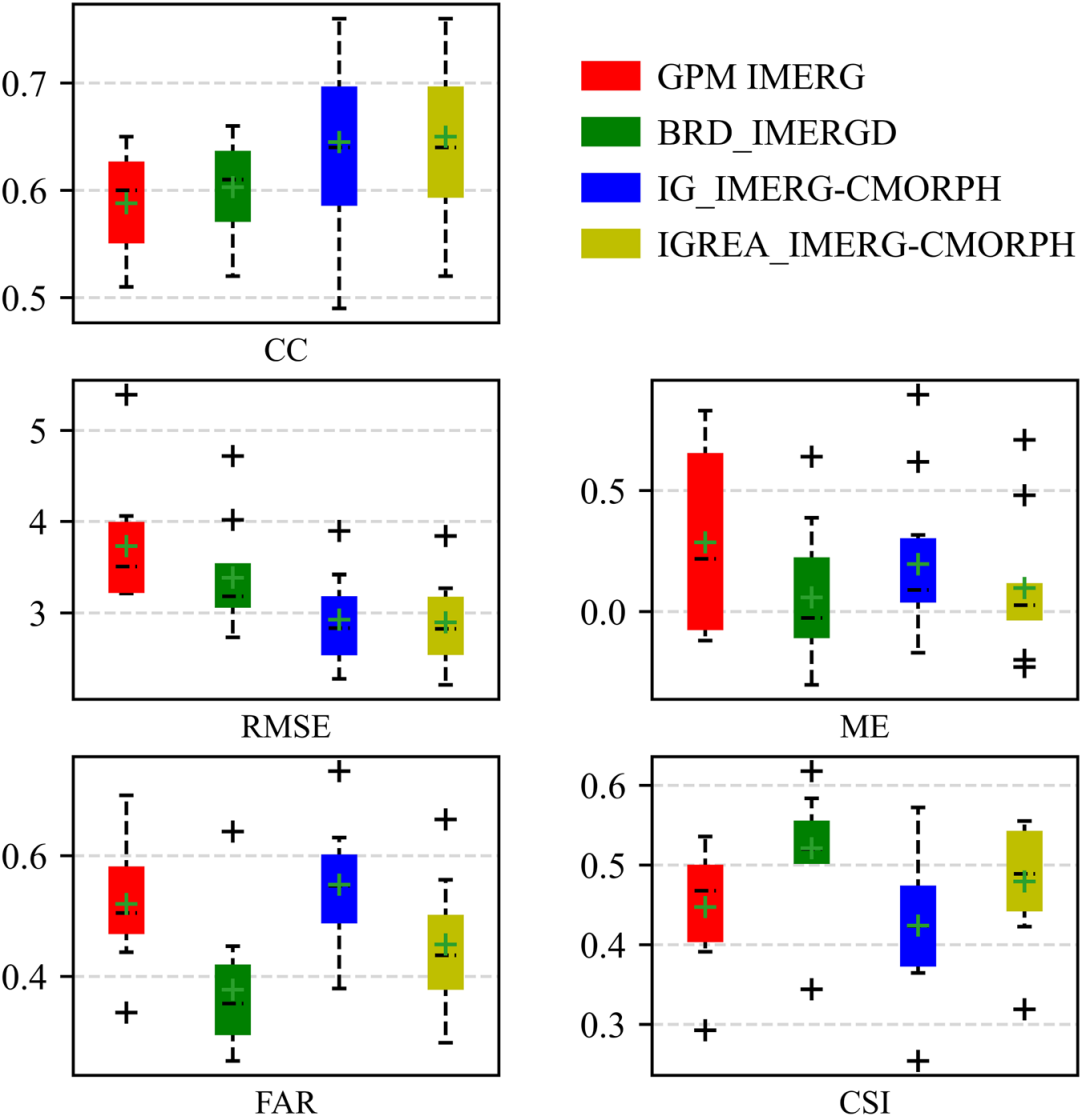
Boxplots of CC, RMSE, ME, FAR and CSI for three reconstructed datasets from 2001 to 2015.

Data at 10 rain gauges (Table 2) was used to validate the performance of three datasets (BRD_IMERG, IGREA_IMERG-CMORPH and IG_IMERG-CMORPH). The evaluation results showed in Fig. 7. For BRD_IMERG and IGREA_IMERG-CMORPH, CCs increased with IMERG (median: 0.60), but the improvement was limited. The median CC of BRD_IMERG (0.61) was less than IGREA_IMERG-CMORPH (0.64). The median MEs were −0.03 and 0.03 mm/day, respectively. The median RMSEs were 3.2 and 2.8 mm/day, respectively. All of them were largely reduced. Obviously, FARs and CSIs also were improved, especially for the corrected product BRD_IMERG. The CC, ME and RMSE of IG_IMERG-CMORPH were close to IGREA_IMERG-CMORPH but CSI and FAR were not good and even worse than IMERG, which proved that the last step of correcting dataset by the precipitation event in the second method was effective. Statistical evaluation revealed that two final products successfully reduced the error and false precipitation event rate.

**Fig. 8:**
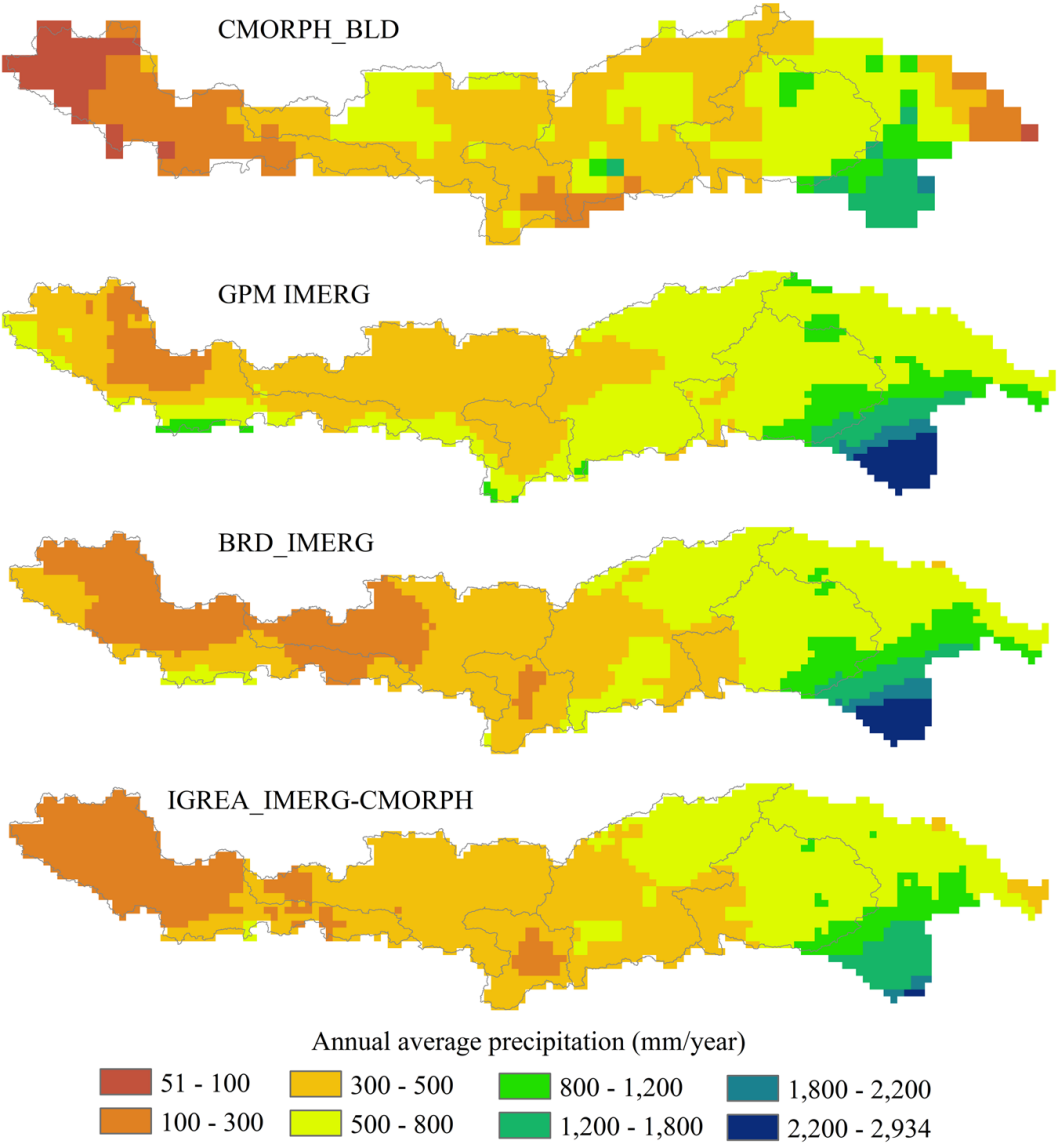
Annual average precipitation of CMORPH_BLD, IMERG, BRD_IMERG and IGREA_IMERG-CMORPH from 2001 to 2015.

**Fig. 9:**
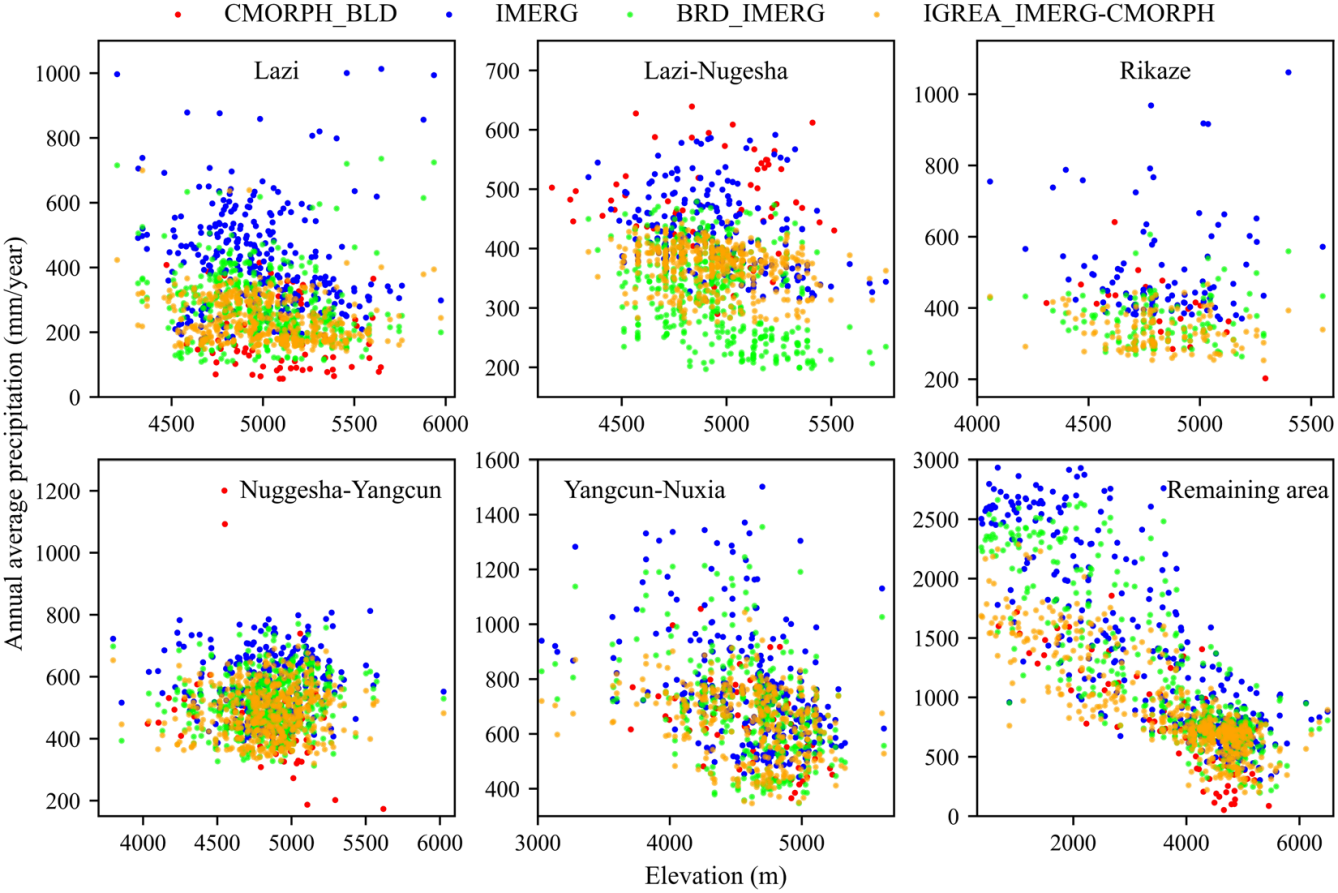
Scatter plots of annual average precipitation with elevation at sub-basins.

**Fig. 10:**
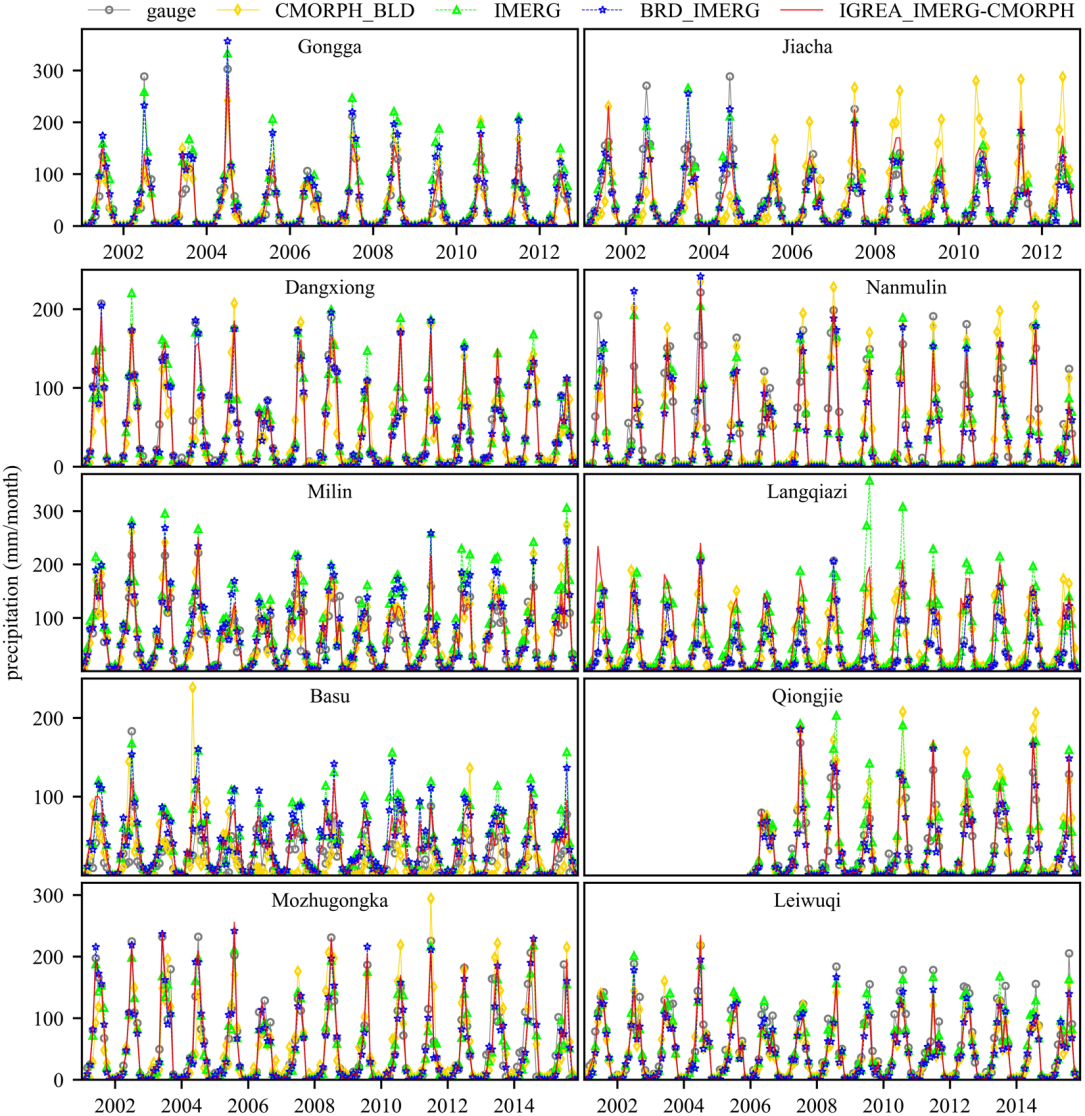
Comparison of precipitation records of rain gauges and corresponding CMORPH_BLD, GPM IMERG, BRD_IMERG and IGREA_IMERG-CMORPH.

**Fig. 11:**
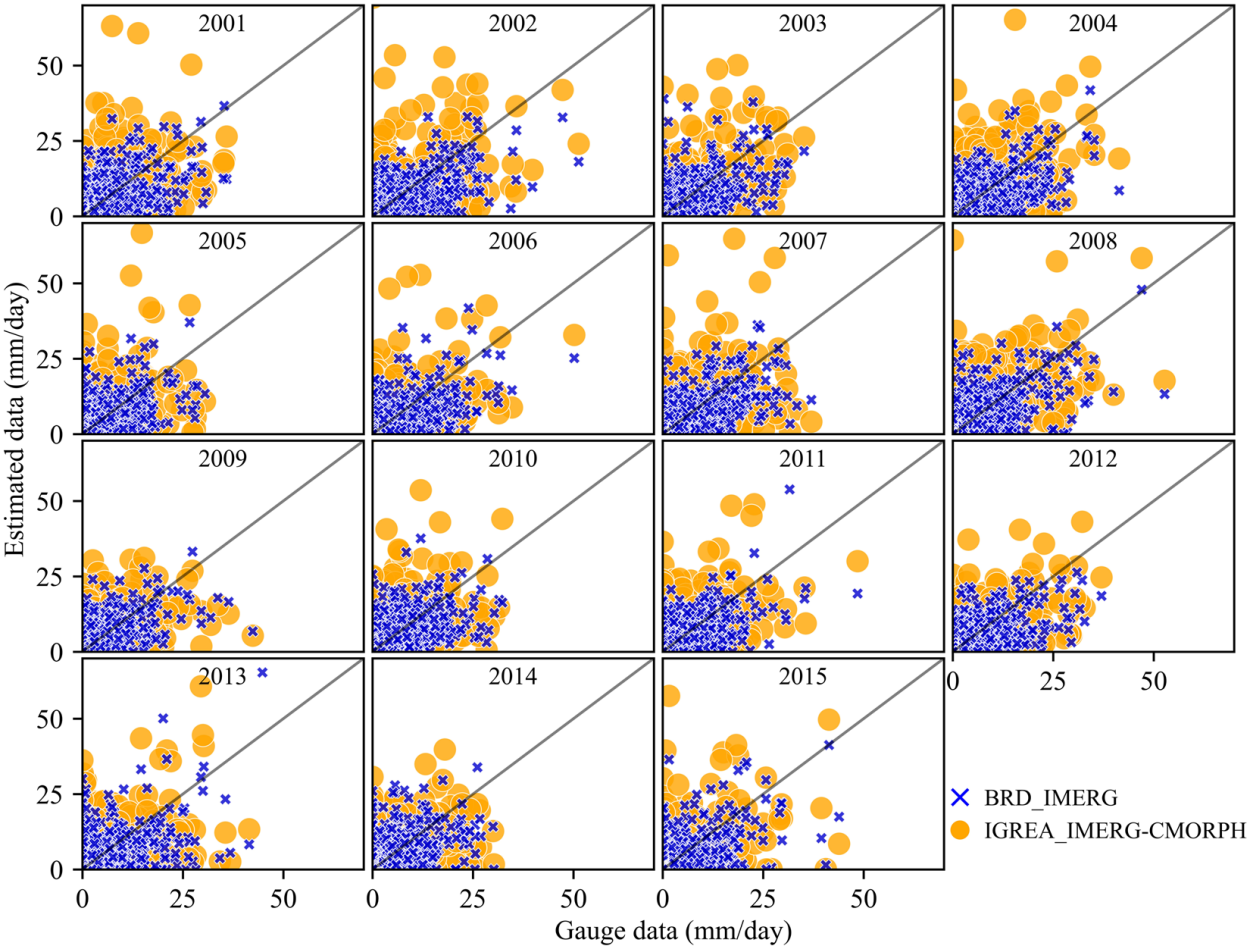
Scatter plots of daily precipitation of rain gauges *v.s*. BRD_IMERG & IGREA_IMERG-CMORPH from 2001 to 2015.

Figures 8 and 9 showed the horizontal and vertical distribution of annual average precipitation (2001~2015) for two input datasets (CMORPH_BLD and IMERG) and two reconstructed datasets (BRD_IMERG and IGREA_IMERG-CMORPH). The annual average precipitation increased from upper reach to lower reach. The downstream area (Remaining area) had a significant decline trend. In addition, Figs. 8 and 9 clearly showed that annual average precipitation of IMERG was usually higher than of CMORPH_BLD, especially in the Lazi, Rikaze and downstream area. The annual average precipitation of BRD_IMERG and IGREA_IMERG-CMORPH were significantly reduced than of IMERG in all sub-basins. Comparison of different precipitation dataset with gauged data (Fig. 10) showed that monthly BRD_IMERG and IGREA_IMERG-CMORPH were closer to the observed precipitation. In Fig. 11, the scatter plots revealed that the daily BRD_IMERG and IGREA_IMERG-CMORPH were in the range of 0 to 65 mm in different years, and there was a small difference with the range of the observed precipitation (0~50 mm). In Fig. 11, BRD_IMERG was more concentrated around the 45° line. It means that the two methods helped to increase the correlation and reduce the error between satellite precipitation products and observations.

### Hydrological evaluation

**Fig. 12:**
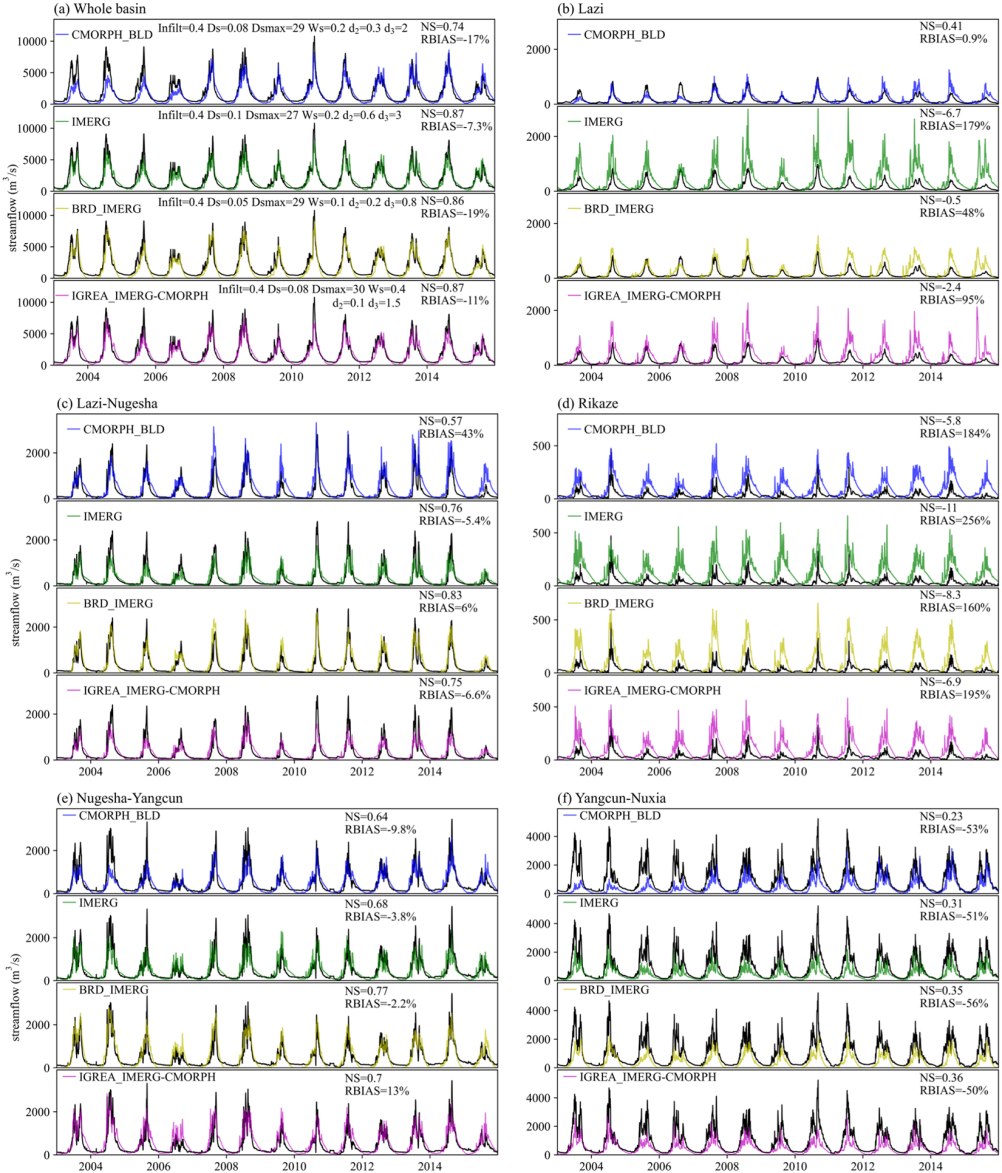
Observed and simulated streamflow by VIC according to four precipitation inputs (CMORPH_BLD, IMERG, BRD_IMERG and IGREA_IMERG-CMORPH) at (**a**) the whole basin and five sub-basins: (**b**) Lazi, (**c**) Lazi-Nugesha, (**d**) Rikaze, (**e**) Nugesha-Yangcun and (**f**) Yangcun-Nuxia. (Note: Black line presents the observed streamflow).

CMORPH_BLD, IMERG, BRD_IMERG and IGREA_IMERG-CMORPH were separately used as the precipitation driver of VIC. The optimal parameter combinations (*Infilt*, *D*_*S*_, *D*_*Smax*_, *W*_*S*_, *d*_2_ and *d*_3_) and simulated streamflow were shown in Fig. 12. NSs and RBIASs in the whole basin were much better than ones in the sub-basins. The simulated streamflow was extremely overestimated in Lazi (RBIAS = 179%) and Rikaze (RBIAS = 256%) sub-basins, and largely underestimated in Yangcun-Nuxia (RBIAS = −51%) sub-basin. NSs of BRD_IMERG and IGREA_IMERG-CMORPH were better than IMERG in sub-basins. CMORPH_BLD had relatively low NS (0.74) and high negative RBIAS (−17%) in the whole basin. Except the Lazi and Rikaze sub-basins, IMERG performed better than CMORPH_BLD in other three sub-basins. The performance of IGREA_IMERG-CMORPH always fell between IMERG and CMORPH_BLD, and further better in the downstream sub-basins. The adjusted dataset BRD_IMERG performed better than IGREA_IMERG-CMORPH in the Lazi, Lazi-Nugesha and Nugesha-Yangcun sub-basins.

The statistical and hydrological results illustrated that BRD_IMERG and IGREA_IMERG-CMORPH would be useful products for analysis of the precipitation with fine resolution in the alpine region. Their advantages and practicalities mainly included: (1) the two products with fine temporal and spatial resolution could meet the research needs at high-altitude regions; (2) the correlation, error and the authenticity degree of precipitation event had been effectively improved; (3) the precipitation estimation was suitable for forcing physical-based hydrological model in the large basin (Yarlung Zangbo River basin).

## Data Availability

The data was processed in Python and ArcGIS. The VIC model code could be downloaded from http://uw-hydro.github.io/.
